# Are Seasonal Acclimation and Genetic Variability of *Lobaria pulmonaria* Relevant for Conservation Translocation? A Case Study Along a Latitudinal Gradient in Italy

**DOI:** 10.3390/plants15091342

**Published:** 2026-04-28

**Authors:** Luca Di Nuzzo, Marta Agostini, Renato Benesperi, Sonia Ravera, Elisabetta Bianchi, Simona Corneti, Silvia Del Vecchio, Luana Francesconi, Gabriele Gheza, Luca Paoli, Juri Nascimbene

**Affiliations:** 1 Department of Biological, Geological and Environmental Science, University of Bologna, 40126 Bologna, Italyluana.francesconi3@unibo.it (L.F.); gabriele.gheza@unibo.it (G.G.); juri.nascimbene@unibo.it (J.N.); 2 Department of Biology, University of Pisa, 56126 Pisa, Italy; marta.agostini@biologia.unipi.it (M.A.); luca.paoli@unipi.it (L.P.); 3 Department of Biology, University of Florence, 50121 Florence, Italy; renato.benesperi@unifi.it (R.B.); e.bianchi@unifi.it (E.B.); 4 Department of Biological, Chemical and Pharmaceutical Sciences and Technologies, University of Palermo, 90123 Palermo, Italy; sonia.ravera@unipa.it

**Keywords:** chlorophyll fluorescence, genetic diversity, threatened lichens, translocation

## Abstract

Understanding the factors that determine the success of lichen translocations is critical for effective conservation of lichen biodiversity. Both physiological acclimation and the genetic structure of source populations can influence conservation outcomes. This study examined seasonal variation in physiological parameters (specific thallus mass—STM, chlorophyll *a* fluorescence—F_V_/F_M_, and chlorophyll content) of *Lobaria pulmonaria* (L.) Hoffm. across one year, selecting three source populations along a latitudinal gradient in Mediterranean forests in Italy. Genetic structure of their mycobiont and photobiont were also characterized. STM differed significantly among populations and seasons, with consistent increases from March to September. In contrast, F_V_/F_M_ remained relatively stable, while chlorophyll content showed the highest values in December. Genetic analyses revealed clear differentiation among populations for both symbionts. These results suggest that *L. pulmonaria* can acclimate physiologically to seasonal environmental changes and highlight the importance of considering local genetic structure when selecting source populations for translocation. Integrating physiological and genetic information provides a robust framework for improving conservation strategies for this species.

## 1. Introduction

Lichens are symbiotic associations involving a heterotrophic partner (the mycobiont) and one or more autotrophic partners (the photobionts), together with associated bacteria and yeasts [1]. This complex organization enables lichens to colonize a wide range of environments, from tundra ecosystems to tropical forests. Despite this broad ecological range, lichens are highly sensitive to human-induced changes, such as anthropogenic climate change, habitat degradation, and pollution [2,3,4,5].

In the context of global change, where multiple stressors may act simultaneously, conservation translocations represent a particularly valuable tool to reduce the negative impacts of human activities on biodiversity [6]. However, their success critically depends on a thorough understanding of species biology, ecological requirements, and clearly defined conservation objectives. The lichen transplant technique (i.e., the transfer of lichen thalli from a remote/control site to a study site) has been used for decades to monitor environmental pollution, e.g., [7,8,9,10,11]. On the other hand, there is a recent growing interest in lichen translocation for conservation purposes [12,13,14], while several key aspects of this approach still require better understanding [14]. An important but often overlooked aspect is the identification of suitable source populations with traits that may enhance establishment success after translocation. Because individuals are moved to environments that differ from their origin, their performance may depend on pre-existing physiological traits and their degree of plasticity [15,16]. Despite its relevance, information on how intraspecific plasticity (particularly the acclimation capacity of source populations) affects translocation success in lichens remains limited.

Understanding the capacity of organisms to acclimate to changing conditions is also fundamental for predicting their responses to ongoing environmental change [17]. For instance, discrepancies between predicted and observed responses to climate change, such as shifts in latitude or elevation, may reflect previously unrecognized acclimation capacity [18]. Integrating this knowledge into conservation practice could substantially improve translocation outcomes.

Lichens are capable, to some extent, of acclimating to environmental variability, including seasonal changes and climate-related stressors [19,20,21,22,23], provided that critical thresholds are not exceeded [24,25]. Acclimation processes can occur at multiple levels, from individual symbiotic partners to the integrated thallus [26]. The photobiont may adjust its photosynthetic apparatus through mechanisms such as increased photoprotective energy dissipation under high irradiance [23,27], modulation of chlorophyll content [21,28], or shifts in temperature-dependent assimilation rates [24]. The mycobiont, in turn, can adjust respiration rates [29], modify thallus thickness in response to seasonal water availability [20], and regulate the production of secondary metabolites or melanins, to balance photoprotection and light transmission [19,22]. In addition, different genotypes or populations within a species may exhibit distinct acclimation capacities, potentially driven by variation in gene expression [30,31]. Widely distributed species are therefore particularly valuable for investigating how populations respond to contrasting environmental conditions [32].

The tripartite epiphytic lichen *Lobaria pulmonaria* (L.) Hoffm. is a well-established model species in ecological and population studies and occurs across a broad range of climatic regions [33]. Due to its long life cycle, it is considered an indicator of undisturbed forest ecosystems and ecological continuity [34,35]. At the same time, it is highly sensitive to air pollution, and its populations have declined markedly over the past century [35]. This species has been widely used to investigate various aspects of lichen biology and evolution [36,37,38] and it has also been tested in translocation studies [36,39,40]. Its wide distribution, ecological sensitivity, and extensive interest in conservation practice make *L. pulmonaria* an ideal model to investigate population-level acclimation across contrasting climatic conditions and to assess how these differences may influence translocation success.

This paper (part of the BioCon*Lobaria* project [41], aimed at improving lichen translocation practices), examines the seasonal variation in specific physiological parameters, namely specific thallus mass (STM), chlorophyll *a* fluorescence and chlorophyll content in source populations from Mediterranean environments. At the same time, the genetic structure of the populations for both the mycobiont *L. pulmonaria* and the green-algal photobiont *Symbiochloris reticulata* (Tschermak-Woess) Skaloud, Friedl, A.Beck & Dal Grande was characterized to account for possible differences in the seasonal pattern due to different genotypes or genetic diversity.

Moreover, even though some previous studies have examined seasonal physiological patterns of *L. pulmonaria* in boreal forests [20,21], data on Mediterranean environments are still scanty.

## 2. Results

### 2.1. Specific Thallus Mass, Chlorophyll a Fluorescence and Chlorophyll Content

A total of 1200 thallus fragments were analyzed across three populations and four seasons. Overall, both spatial and temporal variability contributed to shaping thallus functional traits, although their relative importance differed among parameters (Table 1, Appendix A, Table A1).

**Figure 1:**
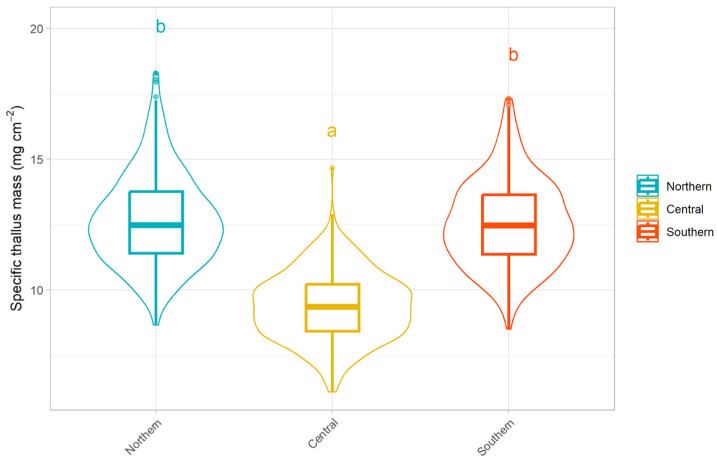
Specific Thallus Mass (mg cm^−2^) for the three populations studied. Different letters indicate significant differences among populations.

Specific thallus mass (STM) differed significantly among populations and seasons (Figure 1). The Central population showed significantly lower STM values (9.39 ± 0.07 mg cm^−2^; *p* < 0.001) compared to the Northern (12.7 ± 0.08 mg cm^−2^) and Southern populations (12.6 ± 0.08 mg cm^−2^), which did not differ from each other.

**Figure 2:**
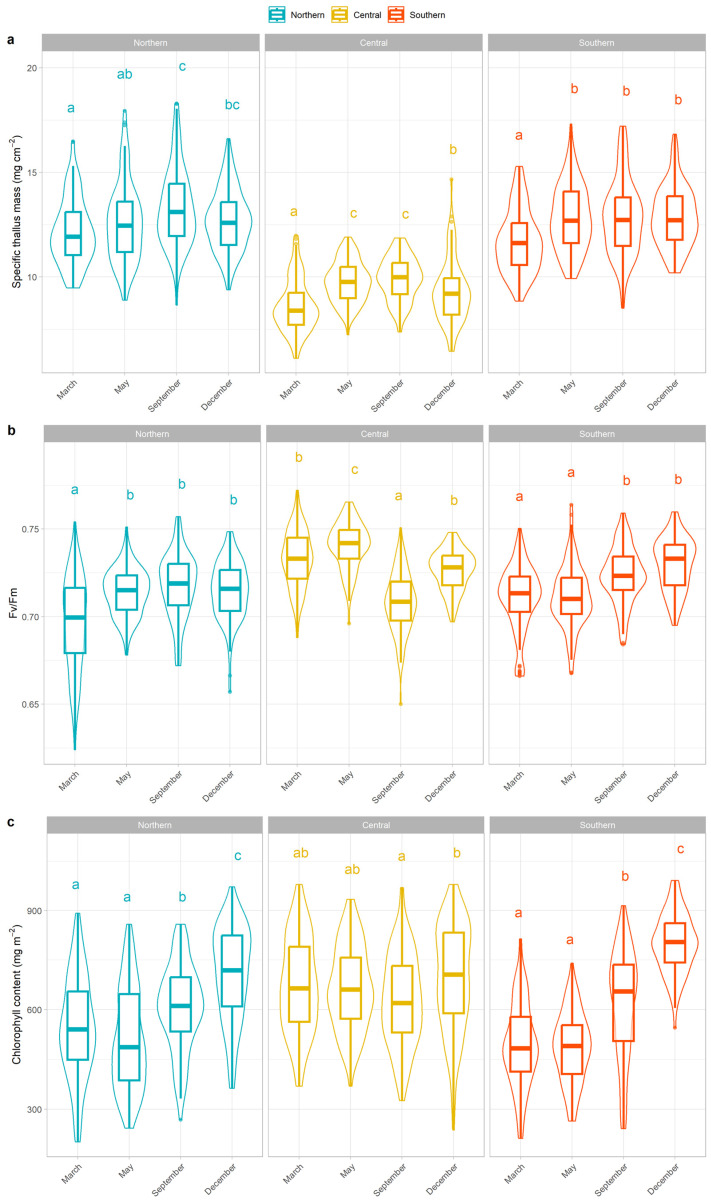
Specific Thallus Mass (mg cm^−2^) (**a**), F_V_/F_M_ (**b**), chlorophyll content (mg m^−2^) (**c**) of the three populations across the four collection seasons. Different letters indicate significant differences among seasons within each population.

In the Northern population, the mean STM increased significantly over the growing season, with higher values in September compared to March and May. Mean STM rose from 12.16 ± 0.16 mg cm^−2^ in March to 13.32 ± 0.19 mg cm^−2^ in September (Table 1, Figure 2). No significant difference was observed between March and May. In December, STM slightly declined, returning to values comparable to early May, although differences were not significant.

The Central population showed a significant increase in STM from March to May and September, followed by a slight, non-significant increase between May and September. In December, STM decreased significantly compared to September (*p* = 0.004; Table 1, Figure 2). The Southern population exhibited a different pattern. Although STM increased significantly from March to May (*p* < 0.01), it remained stable thereafter, with no significant differences among subsequent months (*p* > 0.05; Table 1, Figure 2). 

Chlorophyll *a* fluorescence (F_V_/F_M_) remained consistently high across seasons and populations (0.711 ± 0.001 for the Northern, 0.727 ± 0.001 for the Central and 0.718 ± 0.001 for the Southern populations), with only minor fluctuations. This indicated that the photosynthetic apparatus was largely unaffected by seasonal or site-specific factors, and that thalli maintained a good physiological status throughout the year.

In contrast, chlorophyll content showed clearer seasonal and spatial trends. The higher values observed in the Central population (666 ± 7 mg m^−2^), compared to the Northern and Southern populations (593 ± 8 mg m^−2^ and 601 ± 9 mg m^−2^, respectively) suggested a greater investment in photosynthetic pigments, potentially compensating for its lower STM. In the Northern and Southern populations, chlorophyll content increased during autumn and winter, with a peak in December and reaching minimum values in March (Table 1, Figure 2). A similar trend was observed in the Central population, where the highest values were recorded in December (697 ± 16 mg m^−2^), although differences with March and May were not significant (*p* = 0.603 and *p* = 0.900, respectively; Table 1, Figure 2). 

The low concentrations of potentially toxic elements (Table 2) confirmed that all source populations were representative of unpolluted environments. 

### 2.2. Genetic Structure and Diversity

We analyzed 120 individuals of *L. pulmonaria* from three Italian populations. One monomorphic locus (LPu24) was excluded from further analyses. In addition, one individual from the Northern population was removed due to insufficient amplification quality. For the mycobiont, the highest gene diversity (H) was observed in the Southern population (H = 0.60), followed by the Northern population (H = 0.55), whereas the Central population showed substantially lower diversity (H = 0.34). A similar pattern was observed for allelic richness (Ar), which was highest in the Southern population (7.21), intermediate in the Northern population (6.7), and lowest in the Central population (4.76). The number of multilocus genotypes (G) also varied among populations, with the Southern population showing the highest genotypic diversity (33 MLGs), followed by the Northern (30), and the Central (16) population. All populations possessed private alleles, with the highest number in the Southern population (NAp = 20; Ap = 2.86), followed by the Northern (NAp = 14; Ap = 2.00) and the Central populations (NAp = 11; Ap = 1.57).

Concerning the photobiont, gene diversity was highest in the Northern population (H = 0.76), lowest in the Central population (H = 0.52), and intermediate in the Southern population (H = 0.72). Allelic richness showed a slightly different pattern, with the highest values in the Northern population (5.50), while the Central and Southern populations had similar values (5.18 and 5.20, respectively). The number of multilocus genotypes followed a pattern comparable to the fungal partner, with the highest value in the Southern population (33), followed by the Northern (29) and the Central populations (15). Private alleles were again most frequent in the Southern population (NAp = 19; Ap = 2.71), followed by the Northern (NAp = 17; Ap = 2.43), while the Central population showed the lowest values (NAp = 6; Ap = 0.86). A summary of the data is presented in Table 3.

**Figure A1:**
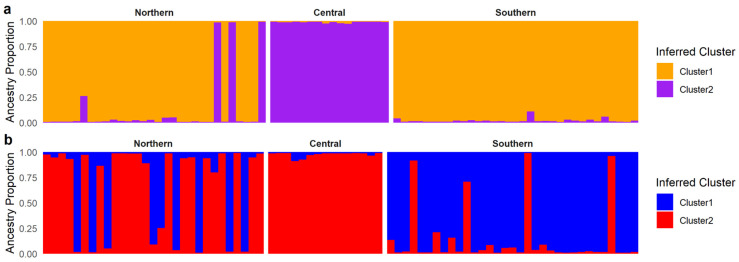
STRUCTURE bar plots for (**a**) *Lobaria pulmonaria* and (**b**) *Symbiochloris reticulata* in the three populations studied. Each gene pool is assigned a distinct color.

**Figure A2:**
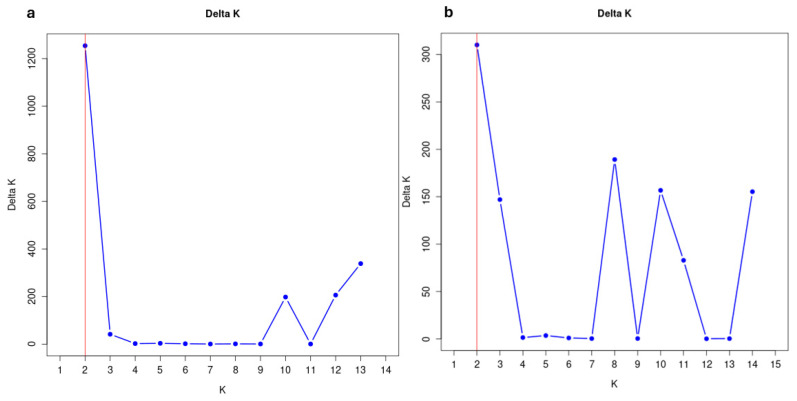
Result of STRUCTURE analyses. Delta K values are plotted for values of K from 1 to 15 in Structure Selector. (**a**) Mycobiont of *L. pulmonaria*. (**b**) Photobiont of *L. pulmonaria*, *S. reticulata*.

Bayesian clustering analysis revealed two distinct genetic clusters (K = 2) for both symbionts (Appendix B, Figure A2). In the fungal partner, all multilocus genotypes (MLGs) from the Central population belonged to a single gene pool, clearly distinct from that of the Southern population. Most MLGs from the Northern population grouped with the Southern cluster, with only three individuals assigned to the Central cluster. A similar pattern was observed for the algal symbiont: all MLGs from the Central population belonged to a single genotype, which was also shared by a subset of individuals from the Northern (9) and Southern (4) populations (Appendix B, Figure A1).

## 3. Discussion

Seasonal variations induced physiological changes in *L. pulmonaria*, allowing it to cope with fluctuating microclimatic conditions throughout the year. Our results suggest that the species modulates both specific thallus mass (STM) and the photosynthetic apparatus in response to seasonal changes, such as those in temperature, light, and precipitation. Although STM differed significantly between populations, the overall annual patterns were consistent across different climatic regions.

STM varied significantly across seasons, supporting the hypothesis that increases in STM are closely related to higher evaporative demands [20]. In lichens, STM represents the dry mass invested per unit thallus area and reflects the balance between biomass accumulation and thallus expansion. Because biomass gain and area growth are not always synchronized, STM can vary seasonally depending on environmental conditions [20,42]. Adjustments in STM can enhance thallus water retention, prolonging periods of metabolic activity during intermittent hydration events [20,43,44].

Under high light and water-limiting conditions, STM tends to increase, and our results are consistent with this pattern. In the studied populations, STM increased from early spring onward, likely in response to progressively lower water availability due to rising temperatures and decreasing precipitation. In Mediterranean climates, the pronounced summer drought often limits hydration opportunities for epiphytic lichens. Increasing STM during this period may therefore represent an acclimation mechanism, allowing the thallus to maintain hydration for longer when water inputs are sporadic. In such conditions, *L. pulmonaria* may rely on early morning humidity to activate metabolic processes and photosynthesize. Growth during these periods may be primarily directed toward increasing thallus thickness, while expansion is more pronounced during the wetter and darker winter months. A thicker thallus may also enhance water retention, creating a functional balance between structural reinforcement during dry periods and expansion during favorable conditions.

Most studies reporting STM values for *L. pulmonaria* in Mediterranean regions describe substantially higher values, typically ranging between approximately 12 and 14 mg cm^−2^ [45,46,47,48]. The lower STM values observed in the Central population of our study suggest that local microclimatic factors, such as higher humidity, reduced irradiance, or more frequent hydration events, may alleviate evaporative stress and allow thalli to maintain structural traits comparable to those in more mesic environments.

Moreover, all populations exhibited similar seasonal changes in STM, both in pattern and magnitude, between March and September (~1.2 mg cm^−2^). This parallel variation is particularly noteworthy because it occurs despite the different absolute STM values among populations. It indicates that, although thallus structure is influenced by local microclimatic conditions, all populations respond in a comparable way to seasonal environmental changes. Interestingly, the minimum and maximum STM values of the Central population in March (8.62 mg cm^−2^) and September (9.91 mg cm^−2^) closely match the range reported by [20] for January and July (8.99 and 10.7 mg cm^−2^, respectively). This finding suggests that relatively low STM values can occur under Mediterranean conditions in the presence of suitable microclimates.

Chlorophyll *a* fluorescence emission showed only slight variation throughout the year. Most fragments displayed F_V_/F_M_ values within the optimal range for *L. pulmonaria* (>0.7), indicating an absence of marked photoinhibition. Only fragments from the Northern population in March and the Central population in May showed statistically significant differences, but these values still fell within the non-stressful range. Our results, obtained in remote areas with suitable conditions for *L. pulmonaria*, are consistent with previous studies reporting no significant seasonal effect on F_V_/F_M_ in *L. pulmonaria* [21], whereas other lichen species (from different environments) do show clear seasonal patterns [22,49]. This stability may result from low light during leafless winter and shading from leaves during periods of higher irradiance, preventing strong photosynthetic inhibition.

In contrast, chlorophyll content exhibited more pronounced seasonal variation. In the Northern and Southern populations, March fragments had the lowest chlorophyll content, which increased throughout the year, with higher values in late autumn. The Central population showed minimal seasonal variation, with only a slight, non-significant increase during winter. Seasonal shifts in chlorophyll content likely represent an acclimation strategy of the photobiont, optimizing light harvesting under changing irradiance, such as seasonal light intensity or canopy closure [28]. In Mediterranean climates, most precipitation and moderate temperatures that favor lichen growth occur primarily in winter, when light availability is lowest. Increasing light-harvesting efficiency during this period is therefore critical for sustaining CO_2_ assimilation and growth.

The significant differences observed between late autumn and March fragments, consistent with previous observations in *L. pulmonaria* [21] and other species [22,50], likely reflect a sharp increase in solar irradiance. The lack of variation between March and May fragments, despite increasing radiation, may be due to tree foliation reducing light exposure. Alternatively, the lower chlorophyll content before spring could reflect seasonal nutrient limitation, which can influence chlorophyll concentration in algal cells under similar light regimes [50,51,52]. While lichens can modulate chlorophyll levels either by adjusting photobiont density or altering pigment concentrations within individual cells [50], the algal population of *L. pulmonaria* remains stable year-round [27,53]. If the nutrient-limitation hypothesis is correct, it would suggest that the observed fluctuations are driven by nutrient-induced physiological shifts within a persistent cell population [53].

Finally, the measured element concentrations in the thalli were consistent with background levels typical of unpolluted environments [13,35,54]. Slightly higher values observed in the population from Northern Italy were limited to soil-associated elements, such as Al and Fe, and do not indicate environmental contamination. This is likely due to the presence of bare soil in the chestnut forest in which thalli were collected, which can resuspend dust that may be adsorbed on the thalli.

These physiological patterns complement the genetic variability detected among populations. Concerning genetic features, all fungal and algal individuals from the Central population belonged to a single genotype. The Southern population also exhibited a single fungal genotype, which was distinct from that of the Central population, whereas the Northern population contained both genotypes. Previous studies have shown that different fungal genotypes of *L. pulmonaria* are often associated with specific microclimatic conditions. For example, distinct fungal gene pools can occupy separate ecological niches even within the same continuous forest landscape [55,56]. Moreover, populations may differ in the magnitude of their genetic responses to changing environmental conditions [30,31]. The Southern population, with the highest gene diversity, allelic richness, and private alleles, may possess greater adaptive potential to cope with seasonal environmental fluctuations, whereas the Central population, with lower genetic diversity, could be more vulnerable to stress. For conservation translocations, these findings emphasize the importance of selecting donor populations based on both genetic richness and physiological resilience. Populations with both high genetic diversity and strong seasonal acclimation are more likely to establish successfully, maintain metabolic activity under fluctuating conditions, and support long-term viability. Preserving co-adapted fungal–algal combinations during translocations is essential, as both partners contribute to seasonal acclimation, sustained growth, and long-term population persistence [38]. The dominance of a single genotype in the Central population may therefore reflect local microclimatic conditions that favor particular genetic lineages. In our study, these genetic differences also corresponded with population-level differences in STM. This raises interesting questions for further research: do populations of these two genotypes consistently exhibit different STM, and if so, is this variation a result of genetic plasticity that enables adaptation to local microclimatic conditions [57], or is it instead a genetically determined trait difference? [58]. Addressing these questions could clarify the relative contributions of genotype and environment to physiological variation in *L. pulmonaria*.

Taken together, these results suggest that translocation activities should consider not only species-specific ecological requirements, but also local population acclimation and potential genotypic differences. This implies that selecting donor populations whose environmental conditions match those of the recipient site may significantly increase establishment success. For example, the conditions under which thalli with lower STM, such as those from the Central population, can survive after translocation may differ from those suitable for thalli with higher STM values. This effect may be further exacerbated during certain periods of the year because of the natural seasonal acclimation of STM observed here. Another important conclusion that emerges from our results is that, given the significant seasonal variation in physiological parameters, Mediterranean translocation programs should preferentially use thalli collected in the same season rather than those collected in different seasons.

Finally, if different genotypes perform better under different conditions, translocating fragments into areas dominated by other genotypes (likely reflecting distinct microclimatic conditions) may reduce translocation success.

## 4. Materials and Methods

### 4.1. Experimental Design

**Figure 3:**
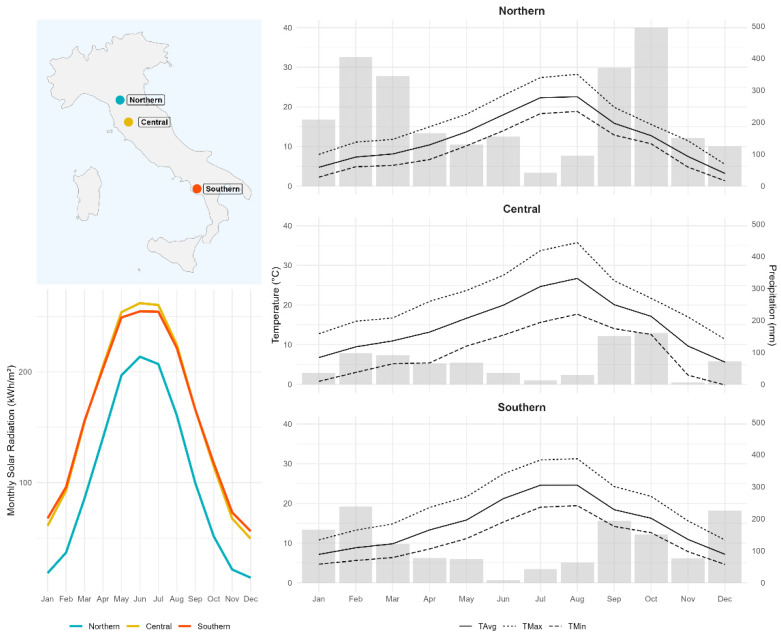
Sampling locations and climatic parameters for each population over the study period (January–December 2024). The right panel shows monthly means of daily minimum air temperature (TMin); daily maximum air temperature (TMax); mean daily air temperature (TAvg).

Three populations of *L. pulmonaria* were selected in remote areas in Mediterranean forests in Italy, as part of the BioCon*Lobaria* project [41]. A key criterion for site selection was the total population size: only large, well-established populations were considered to ensure that thallus collection for the translocation experiment would not compromise the long-term vitality or reproductive potential of the source communities. The populations were selected according to a latitudinal gradient (Figure 3). The Northern population was located in a chestnut (*Castanea sativa* Mill.) forest near Pianaccio, municipality of Lizzano in Belvedere (BO), Emilia-Romagna, Italy (WGS84: N 44.134245°, E 10.870254°, 911 m a.s.l.). The area is characterized by a warm-humid continental climate, with relatively warm summers and cold winters. Precipitation is generally well distributed throughout the year, and was 2681 mm in 2024, with an average annual temperature of around 12.2 °C. The Central population was located in a mixed oak-dominated (*Quercus pubescens* Willd. and *Quercus cerris* L.) forest (WGS84: N 43.182556°, E 11.365385°), near Crevole, municipality of Murlo (SI), Tuscany, Italy. Finally, the Southern population was located in an oak-dominated (*Q. cerris*) forest (WGS84: N 40.298816°, E 15.328061°), near Laurino (SA), Campania, Italy. The Central and Southern areas (altitude 279 m and 868 m, respectively) are generally characterized by warm-summer Mediterranean climate, with hot and dry summers and mild and rainy winters. Precipitation, mainly concentrated in autumn and spring, was around 819 mm and 1438 mm in 2024 for Central and Southern areas, respectively, and the mean annual temperature was approximately 15.1 °C and 14.8 °C. The surveyed areas were comparable in terms of surface area (around 1 ha) and number of trees colonized by *L. pulmonaria* (>60).

For the specific goals of this work, every three months (from March 2024 to December 2024), 100 thallus fragments with 3–6 cm diameter and with presence of vegetative diaspores were randomly selected for evaluating physiological parameters. All fragments were collected from large thalli to avoid significant damage to the parent thalli. In all three populations, the presence of fertile thalli with apothecia was also recorded.

Meteorological data were obtained from the nearest weather stations (2–10 km from the study sites). Potential solar radiation (insisting over each population) was estimated by assuming clear-sky conditions through the r.sun.daily module in GRASS GIS 8.4, via the rgrass R package [59]. We used the Copernicus Global DEM with 30 m resolution obtained using the elevatr [60] package in R to calculate slope and aspect with terra package [61]; while terrain horizon was computed by the r.horizon GRASS GIS module. Data calculated by summing direct and diffuse radiation were monthly aggregated and are shown in Figure 3.

### 4.2. Chlorophyll a Fluorescence Emission and Chlorophyll Content

Chlorophyll *a* fluorescence emission was analyzed using a Handy PEA fluorimeter (Plant Efficiency Analyzer, Hansatech Ltd., Norfolk, UK). After collection, thalli were sprayed and left hydrated for an entire night. Before measurement, each thallus fragment was dark-adapted for 10 min using a clip randomly positioned on its surface. The sample was then exposed to a saturating pulse of red light (650 nm, intensity up to 1800 μmol photons m^−2^ s^−1^) for one second. Three fluorescence induction curves were recorded for each thallus fragment for one second. The resulting curves were analyzed using the JIP-test approach [62,63], which allows raw fluorescence signals to be transformed into a set of biophysical parameters. These parameters provide quantitative information on energy fluxes, photosynthetic performance, and the structural and functional condition of the photosynthetic apparatus. The physiological status of the samples was initially evaluated through the maximum quantum yield of primary photochemistry, calculated as φPo = (F_M_ − F_0_)/F_M_ = F_V_/F_M_. In addition, the chlorophyll content of the thalli was measured by a Chlorophyll Content Meter-300 (Opti-Sciences CCM-300, Hudson, NH, USA), which, based on reflectance and/or absorbance of radiation by chlorophyll molecules, estimates the chlorophyll content expressed as m^2^ of biological material (mg m^−2^).

### 4.3. Specific Thallus Mass

To measure the Specific Thallus Mass (STM), fully hydrated samples with distilled water were flattened and scanned using a high-resolution color scanner (600 dpi; EPSON Perfection V370 Photo, Seiko Epson Co., Nagano, Japan). The ImageJ software version 1.54 was used to determine the hydrated fragment area through direct measurements of the fragment size on the images obtained from high-resolution 2D scanning. To estimate dry mass, fragments were first left to dry at room temperature for at least 24 h, then kept in a silica gel desiccator for additional 24 h. For each measurement set, three thalli were sacrificed to determine the relationship between air-dried and oven-dried mass. These samples were initially weighed following the same procedure used for the remaining thalli and then oven-dried at 60 °C for 72 h to obtain their constant dry weight. The difference between the initial dry weight and the oven-dried weight was used to estimate residual water content. A correction factor derived from these sacrificed thalli was then applied to convert the air-dried mass of all samples into dry mass (DM).

### 4.4. Element Concentrations

To prepare each sample, approximately 200 mg of lichen material was carefully selected using plastic tweezers. Air-dried samples were manually cleaned (without washing) to remove extraneous material such as bark pieces, soil particles, and moss fragments. The cleaned material was then milled under liquid nitrogen using a ceramic mortar and pestle, and mineralized in triplicate with a mixture of 3 mL of 70% HNO_3_, 0.2 mL of 60% HF and 0.5 mL of 30% H_2_O_2_. Microwave digestion was performed using a Milestone Ethos 900 system (Milestone Srl, Sorisole, Italy). The following elements of toxicological concern (Al, Cd, Cr, Cu, Fe, Hg, Ni, Pb, S, Sb, Zn) were quantified by inductively coupled plasma mass spectrometry (ICP-MS, PerkinElmer SCIEX ELAN 6100, PerkinElmer SCIEX, Waltham, MA, USA) and concentrations were expressed on dry weight basis. Analytical quality was checked with the Standard Reference Material IAEA-336 (lichen) [64] for Al, Cd, Cr, Fe, Pb, Sb, Zn, or alternatively GBW-07604 (Poplar leaves) [65] for Cu, Hg, Ni and S with recoveries ranging from 90 to 112%. The precision of analysis (range of variation within 11%) was estimated by the variation coefficient of three independent replicates.

### 4.5. DNA Extraction and Microsatellite Genotyping

For DNA extraction and microsatellite genotyping, 40 thalli from each source population were selected, being randomly sampled from the bark of dominant trees in each source area, irrespective of cardinal exposure and distance from soil. Approximately 10 mg of thallus tissue was used for DNA extraction using the E.Z.N.A.^®^ Plant & Fungal DNA Kit (Omega Bio-tek., Inc., Norcross, GA, USA), following the manufacturer’s protocol. Total DNA was amplified using eight commonly used fungus-specific microsatellites (LPu03, LPu09, LPu15, LPu23, LPu24, LPu25, LPu28, MS4; [55,66,67]) with fluorescently labeled primers, according to the protocols reported in the cited studies. Seven commonly used algal microsatellites (LPh1-LPh7, [68,69]) were also amplified. For specific PCR conditions, see Appendix A. The amplified fragments were subsequently analyzed using a 3730xl DNA Analyzer (Applied Biosystems, Waltham, MA, USA) at the Macrogen facility. Genotyping was performed using GENEMAPPER 4 (Life Technologies, Carlsbad, CA, USA).

### 4.6. Data Analysis

For chlorophyll content and chlorophyll *a* fluorescence emission, over each thallus three measurements were carried out and averaged. To test the effect of different populations and seasonality on STM, chlorophyll content and chlorophyll *a* fluorescence, we fitted a linear model with population, season and their interactions. STM was square-rooted to meet normality. The potential quantum yield of primary photochemistry (F_V_/F_M_) was analyzed using generalized linear models with a beta error distribution. Because variability in F_V_/F_M_ differed among seasons, the dispersion parameter of the beta distribution was allowed to vary as a function of season. Models were fitted using the R package glmmTMB version 1.1.14 [70]. The estimated marginal means (EMMs) were computed separately for the effect population and seasons. Pairwise comparisons were adjusted using Tukey’s method, and differences among groups were generated using compact letter displays (CLDs) using the package emmeans [71] and multcomp [72]. Model diagnostics were investigated using the DHARMa package version 0.4.7 [73].

To evaluate the genetic pattern of the populations, all clones were removed and only different multilocus genotypes were maintained, reducing the overall dataset to 79 individuals for the mycobiont and 77 for the photobiont. A Bayesian analysis for the population structure was run using STRUCTURE 2.3.4 [74] separately for the fungal and the algal symbionts. The most likely number of clusters [75] was assigned using the method in the online platform Structure Selector web tool [76]. Analyses considered K values from 1 to 15, and delta K values (representing the rate of change in log-likelihood between successive K values), were used to identify the most probable number of clusters. The whole dataset, including clones, was used to estimate several metrics of genetic diversity for each population for the algal and fungal partners separately. Allelic richness (Ar) was estimated using the function *allelic.richness* of the hierfstat package version 0.5-11 [77]. Basic population genetic statistics, such as number of multilocus genotypes, Nei’s gene diversity, were computed using the function *poppr*. Private alleles were identified using the function *private_alleles* implemented in the poppr package version 2.9.8 [78]. For each population, the number of private alleles (NAp) was calculated as the count of alleles occurring exclusively in that population. The proportion of private alleles (Ap) was then obtained by dividing the number of private alleles by the total number of loci analyzed. All analyses were performed using R version 4.5.1 [79].

## 5. Conclusions

*Lobaria pulmonaria* showed seasonal acclimation in remote Mediterranean forests, involving both fungal and algal partners, allowing populations to maintain physiological functionality under varying environmental conditions. Despite differences in absolute trait values among populations, the overall seasonal trajectories of key physiological parameters (chlorophyll *a* fluorescence and chlorophyll content) were remarkably consistent along the latitudinal gradient, indicating a shared acclimation strategy across macroclimatic contexts.

At the same time, population-specific differences in structural traits (e.g., STM) and the distinct genetic composition of both symbionts highlight the role of local environmental conditions in shaping functional and genetic variability. The dominance of specific genotypes within populations, coupled with their association with physiological traits, suggests that acclimation capacity may be partly constrained or facilitated by genetic background.

These findings have direct implications for conservation. The ability of *L. pulmonaria* to acclimate across seasons supports its potential resilience to environmental variability; however, the observed genetic differentiation among populations indicates that source selection in translocation programs should not be based solely on ecological matching, but also on preserving locally adapted or co-adapted symbiotic combinations.

Overall, this work suggests that integrating physiological plasticity with population genetic structure provides a more comprehensive framework for understanding species responses to environmental change. Such an approach is essential for improving the effectiveness of translocation strategies and for ensuring the long-term conservation of lichen populations under ongoing global change.

## Figures and Tables

**Table 1:** Summary statistics of Specific Thallus Mass (STM), chlorophyll content and the potential quantum yield of primary photochemistry (F_V_/F_M_) for the three populations considered in the four seasons of collection.

Population	Season	STM (mg cm^−2^)	Chlorophyll (mg m^−2^)	F_V_/F_M_
Northern	March	12.16 ± 0.16	547 ± 15	0.696 ± 0.003
	May	12.58 ± 0.19	510 ± 15	0.714 ± 0.001
	September	13.32 ± 0.19	606 ± 13	0.717 ± 0.002
	December	12.73 ± 0.15	709 ± 14	0.715 ± 0.002
Central	March	8.62 ± 0.12	673 ± 14	0.733 ± 0.002
	May	9.76 ± 0.10	665 ± 13	0.740 ± 0.001
	September	9.91 ± 0.10	629 ± 14	0.708 ± 0.002
	December	9.29 ± 0.15	697 ± 16	0.726 ± 0.001
Southern	March	11.69 ± 0.15	493 ± 13	0.711 ± 0.002
	May	12.83 ± 0.16	487 ± 11	0.711 ± 0.002
	September	12.84 ± 0.18	621 ± 16	0.723 ± 0.002
	December	12.87 ± 0.14	801 ± 9	0.728 ± 0.002

Values are expressed as Mean ± SE.

**Table 2:** Content of potentially toxic elements (µg/g dw) in *Lobaria pulmonaria* from source populations and comparison with a previous study in remote forests in Italy.

Population	Al	Cd	Cr	Cu	Fe	Hg	Ni	Pb	S	Sb	Zn
Northern Italy	1087	0.212	3.76	7.60	1549	0.093	1.92	1.67	1404	0.088	39.2
Central Italy	463	0.113	1.22	7.59	441	0.044	0.96	0.80	996	0.079	19.5
Southern Italy	415	0.229	0.94	5.78	427	0.047	0.54	0.87	1040	0.059	19.1
Average ± SD	655 ± 375	0.185 ± 0.063	1.98 ± 1.56	6.99 ± 1.05	806 ± 644	0.061 ± 0.027	1.14 ± 0.71	1.11 ± 0.48	1147 ± 224	0.075 ± 0.015	25.9 ± 11.5
Central Italy [13]	536	0.66	1.7	6.0	-	-	1.4	1.3	1042	0.08	19

**Table 3:** Summary statistics of the genetic diversity of the three populations studied for the fungal and algal partners separately. G: number of multilocus genotypes; H: Nei’s unbiased gene diversity; Ar: mean within population allelic richness; Ap: mean number of private alleles from all loci; NAp: number of alleles privates.

Population	Season	G	H	Ar	Ap	NAp
Mycobiont	Northern	30	0.55	6.69	2.00	14
	Central	16	0.35	4.76	1.57	11
	Southern	33	0.60	7.21	2.86	20
Photobiont	Northern	29	0.76	7.71	2.43	17
	Central	15	0.52	5.62	0.86	6
	Southern	33	0.72	7.76	2.71	19

## Data Availability

The raw data supporting the conclusions of this article will be made available by the authors on request.

## Appendix A

### PCR Conditions

We divided the amplification of the primer for the mycobiont into three duplex reactions and two singleplex reactions. Specifically, we amplified LPu03 and LPu09, LPu23 and MS4, and LPu24 and LPu28 in duplex reactions, while LPu15 and LPu25 were amplified in singleplex. For LPu03, LPu09 and LPu15 we used 400HD as the internal size standard, while for LPu23, LPu24, LPu25, LPu28 and MS4 we used LIZ500. The PCR reactions were performed using 1 × GoTaq^®^ Flexi PCR buffer (Promega, Beijing, China), 200 µM dNTPs, and 0.125 U GoTaq^®^ G2 Flexi DNA polymerase (Promega). For the duplex reactions LPu03–LPu09 and LPu23–MS4, and the singleplex LPu15, we used a MgCl_2_ concentration of 2 mM, while for the duplex LPu24–LPu28 we used 2.5 mM and for the singleplex LPu25 we used 1.5 mM. The PCR cycle was as follows: an initial denaturation of 30 s at 95 °C, followed by 30 cycles of 30 s at 95 °C, 30 s at 54–55 °C (depending on the primer), and 1 min at 72 °C, with a final extension step of 5 min at 72 °C. The annealing temperature was set at 54 °C for LPu15, 54.4 °C for LPu23, LPu24, LPu25 and MS4, and 55 °C for LPu03 and LPu09. Primer concentrations were optimized as follows: LPu03, LPu09, LPu23, and LPu24 at 0.2 µM; MS4 at 0.25 µM; LPu28 at 0.3 µM; and LPu25 and LPu15 at 0.4 µM. At the same time, we amplified seven alga-specific microsatellites from the photobiont (LPh1, LPh2, LPh3, LPh4, LPh5, LPh6, LPh7; [66]). Two multiplex PCR reactions were performed: one including LPh2, LPh4 and LPh5, and one including LPh1, LPh3, LPh6 and LPh7. The forward primers were fluorescently labeled as follows: LPh1 (JOE), LPh2 (FAM), LPh3 (TAMRA), LPh4 (TAMRA), LPh5 (ATTO565), LPh6 (FAM) and LPh7 (JOE). The PCR reactions were set up using 1 × GoTaq^®^ Flexi PCR buffer (Promega), 2 mM of MgCl_2_ 300 µM dNTPs, and 0.150 U GoTaq^®^ G2 Flexi DNA polymerase (Promega). The PCR cycle was as follows: an initial denaturation of 30 s at 95 °C, followed by 30 cycles of 30 s at 95 °C, 30 s at 57 °C, and 1 min at 72 °C, with a final extension step of 5 min at 72 °C. Primer concentrations were optimized as follows: LPh2, Lph3, LPh4 0.2 µM; LPh5 and LPh7 at 0.3 µM and Lh4 at 0.4 µM.

**Table A1:** Anova Type III table of the three models testing the influence of Season, Population and their interaction on the studied physiological parameters: specific thallus mass (STM), potential quantum yield of primary photochemistry (F_V_/F_M_), chlorophyll content (Chl).

Model	Term	Chisq	df	*p* Value
	Season	28.23	3	0.000
STM	Population	379.98	2	0.000
	Season:Population	18.55	6	0.005
	Population	153.77	2	0.000
F_V_/F_M_	Season	69.74	3	0.000
	Population:Season	301.59	6	0.000
	Population	91.93	2	0.000
Chl	Season	97.05	3	0.000
	Population:Season	155.07	6	0.000
